# Vibration Characteristics of Magnetostrictive Composite Cantilever Resonator with Nonlocal Effect

**DOI:** 10.3390/s24165390

**Published:** 2024-08-21

**Authors:** Yan Xu, Xinchun Shang, Ke Xu

**Affiliations:** 1 National Center for Materials Service Safety, University of Science and Technology Beijing, Beijing 100083, China; shangxc@ustb.edu.cn; 2 School of Mathematics and Physics, University of Science and Technology Beijing, Beijing 100083, China; 3 Collaborative Innovation Center of Steel Technology, University of Science and Technology Beijing, Beijing 100083, China; xuke@ustb.edu.cn

**Keywords:** magnetostrictive resonator, nonlocal theory, composite cantilever, nature frequency, vibration mode

## Abstract

Taking the nonlocal effect into account, the vibration governing differential equation and boundary conditions of a magnetostrictive composite cantilever resonator were established based on the Euler magnetoelastic beam theory. The frequency equation and vibration mode function of the composite cantilever were obtained by means of the separation of variables method and the analytic solution of ordinary differential equations. The lateral deflection, vibration governing equations, and boundary conditions were nondimensionalized. Furthermore, the natural frequency and modal function of the composite beam were quantitatively analyzed with different nonlocal parameters and transverse geometry dimensions using numerical examples. Compared with the results without considering the nonlocal effect, the influence of the nonlocal effect on the vibration characteristics was analyzed. The numerical results show that the frequency shift and frequency band narrowing of the magnetostrictive cantilever resonator are induced by nonlocal effects. In particular, the high-frequency vibration characteristics, such as vibration amplitude and modal node of the composite beam, are significantly affected. These analysis results can provide a reference for the functional design and optimization of magnetostrictive resonators.

## 1. Introduction

Smart materials are materials that have both intrinsic and extrinsic capabilities to respond to stimuli and environmental changes and activate their functions in response to these changes. Magnetostrictive materials with good magneto-mechanical coupling properties undergo dimensional changes when exposed to a magnetic field, and conversely, they can also effectively transform the alternating magnetic energy in the environment into mechanical vibration through this magnetostrictive effect. As an important component device in micro-electro-mechanical systems (MEMS), magnetostrictive resonators are widely used in actuators, sensors, energy collectors, frequency controllers, and logic switches [1,2,3,4]. High-performance MEMS require the resonator to have the advantages of high sensitivity, high precision, and low energy consumption when operating. Due to the complexity and miniaturization of resonators, higher requirements are put forward for their vibration performance. For example, the operating bandwidth is wider, the energy density is higher, and the driving voltage is lower [5,6,7,8].

Therefore, it is important to accurately predict the vibration characteristics of microsized magnetostrictive resonators to improve the design and use of resonator devices.

Magnetostrictive resonators are divided in structure into two major categories, rod-type and beam-type, which are mostly made into cantilevers. For the longitudinal vibration of rod-type resonators, relative experiments, numerical simulations, and theoretical analyses are available in the references [9,10,11]. Recently, beam-type magnetostrictive resonators with layered composite structures have attracted much attention because of their multi-interface physical field effects, such as magnetomechanics and magnetoelectric. Yu et al. [12] used Kirchhoff theory to simulate the surface effect by applying the spring force on the boundary and analyzed the influence of electromechanical resonance characteristics of the nanocantilever beam device in MEMSs. Jia et al. [13] proposed a nonlinear magnetomechanical coupling model at low magnetic fields using Gibbs free energy method, and the predicted results were in good agreement with the experimental data under given prestress conditions. Reddy et al. [14] obtained an analytical solution to the vibration problem of giant magnetostrictive laminated simply supported beams based on Hamilton's principle. Xu and Shang [15] used high-order shear deformation theory and linear coupled magnetoelastic constitutive relations to construct the vibration governing equation of a magnetostrictive composite cantilever actuator and presented the influence of geometrical, material parameters, and excitation frequency on the structure's magneto-mechanical coupling coefficient in detail. Xu and Shang [16] applied the nonlinear constitutive relation of magnetostrictive materials to obtain the vibration differential equation of a double-layer cantilever beam using Hamilton's principle and analyzed frequency-doubling dynamic characteristics of the amplitude–frequency curve and deflection response under periodic magnetic field excitation. At present, the theoretical research of magnetostrictive resonator model building is almost entirely based on classical beam or plate theory, but the mathematical model building of micro-devices rarely considers or ignores the influence of microscale on the structural vibration characteristics.

As early as 1994, Quandt et al. [17] studied and designed a double-layer thin-film cantilever-type magnetostrictive actuator and obtained the relationship curve between deflection and magnetic field strength through experiments. In recent years, researchers [8,18,19] designed magnetoelectric composite resonators with different structural forms and observed the effects of frequency doubling, mixing, and resonance frequency shifts of resonators under a magnetic field through experiments. Using the finite element method, the sensing characteristics of the cantilever resonator under different vibration modes [20] and the design optimization of the device were analyzed [21], as well as the influence of the magnetic field on the resonant frequency [22]. In addition, magnetostrictive resonators have also become a research focus of scholars in biosensing technology due to their advantages in wireless detection, simple operation, high sensitivity, and fast detection speed [23,24].

Experimental results showed the size-dependent effect of the magnetostrictive material on a small scale (i.e., micro/nano-scale), which plays an important role in designing miniaturized smart devices [25,26]. Therefore, it is necessary to understand the vibration behavior of magnetostrictive micro resonators by considering microscale theoretical models. So far, continuum mechanics is commonly used to describe the size-dependent effect in microstructures [27,28]. Based on previous studies [15,16], this paper introduced microscale parameters into the material constitutive equation via nonlocal elasticity theory and established a vibration control model of a magnetostrictive composite cantilever beam. The effect of microscale parameters on the vibration characteristics of the resonator is further studied by numerical calculation.

## 2. Theoretical Foundation and Modeling

**Figure 1:**
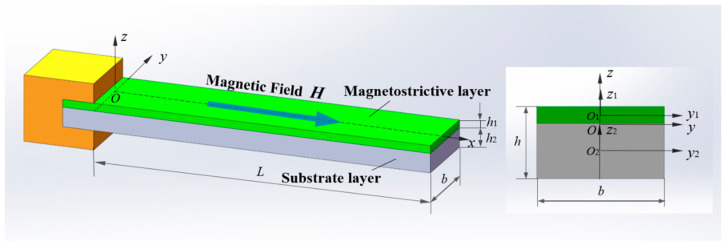
Schematic diagram of geometric dimensions and coordinates for the composite cantilever beam.

We consider the vibration of a rectangular double-layer composite cantilever beam generated by the uniformly distributed horizontal magnetic field *H*, as depicted in Figure 1. The length of the beam is *L*, the width is *b*, and the thickness is *h*. Layer one and layer two refer to the magnetostrictive layer and the elastic substrate layer, respectively. A Cartesian coordinate system *Oxz* is attached to the beam with *z* along the beam thickness direction and *x*-axis on the interface of the magnetostrictive layer and the elastic substrate layer. The elastic modulus is denoted as *E_i_*, the shear elastic modulus as *G_i_*, Poisson’s ratio as *υ_i_*, the density as *ρ_i_*, the thickness as *h_i_*, the cross-sectional area *A_i_*, and the moment of inertia to the *y_i_* axis as $I_{yi}=bh_{i}^{3}/12$ for the *i*-th layer of the composite cantilever beam (*i* = 1, 2), where *i* = 1 and *i* = 2 represent the magnetostrictive layer and the base layer, respectively.

Different from classical elasticity theory, the nonlocal continuum theory is capable of capturing the size-dependent effect in magnetostrictive microstructures. In nonlocal theory, the stress at an arbitrary point of the magnetostrictive microstructure is a function of the strain and magnetic field at all points in the structure. Thus, based on the nonlocal elasticity theory of Eringen [29], the magnetoelastic constitutive relation of a one-dimensional composite beam can be written in the following form:

(1) $$\left[1-{\left(e_{i}a_{i}\right)}^{2}\frac{\partial^{2}}{\partial x^{2}}\right]\sigma_{x}^{\left(i\right)}=E_{i}\varepsilon_{x}^{\left(i\right)}-E_{i}d_{i}H\left(t\right)\left(i=1,\;2\right)$$

where $\sigma_{x}$, $\varepsilon_{x}$ and $H\left(t\right)$ are the stress, strain, and magnetic field, respectively. It is emphasized that *ea* is the nonlocal parameter, which represents the influence of small scale on the behavior of microstructure, where *e* is a given material constant and can be determined by molecular dynamics simulations, experiments, or other computational methods [30] and *a* depends on the internal microstructure size of the material such as C-C bond length, lattice spacing, and particle distance. Furthermore, *d* is the magnetostrictive coefficient, as the elastic substrate layer is not influenced by the magnetic field, where the parameter *d*_2_ = 0.

Based on the Euler–Bernoulli beam theory, the displacements of the composite beam can be assumed to be as follows:

(2) $$\left\{\begin{array}{l} u_{x}^{\left(i\right)}(x,z,t)=u(x,t)-z\frac{\partial w\left(x,t\right)}{\partial x}\\ u_{z}^{\left(i\right)}(x,z,t)=w(x,t) \end{array}\right.(i=1,2)$$

where *u*(*x*, *t*) and *w*(*x*, *t*) are the longitudinal displacement and the deflection at the interface (*z* = 0), respectively.

The axial force, shear force and bending moment of the layer *i*th cross section are denoted by $F_{N}^{\left(i\right)}$, $F_{S}^{\left(i\right)}$ and $M^{\left(i\right)}$ (*i* = 1, 2), respectively. $F_{N}=F_{N}^{\left(1\right)}+F_{N}^{\left(2\right)}$ and $M=M^{\left(1\right)}+M^{\left(2\right)}$ are the total transverse shear force and the total bending moment acting on the whole cross section, and the torque of the axial force on the *y*-axis is omitted. Ignoring the horizontal inertial force and inertial couple, we can obtain the internal force dynamic balance equation:

(3) $$\frac{\partial F_{N}^{\left(1\right)}}{\partial x}+\frac{\partial F_{N}^{\left(2\right)}}{\partial x}=0$$

(4) $$\frac{\partial F_{S}}{\partial x}=(\rho_{1}A_{1}+\rho_{2}A_{2})\frac{\partial^{2}w}{\partial t^{2}}$$

(5) $$\frac{\partial M}{\partial x}=F_{S}$$

It is assumed that the nonlocal length parameters of the upper and lower layers of the composite beam are the same, namely $\mu ={\left(e_{1}a_{1}\right)}^{2}={\left(e_{2}a_{2}\right)}^{2}$. Substituting the geometric relation $\varepsilon_{x}^{\left(i\right)}=\partial u_{x}^{\left(i\right)}/\partial x$ into Equation (1), and using Equation (2), the axial stress of each layer can be expressed as follows:

(6) $$\left[1-\mu \frac{\partial^{2}}{\partial x^{2}}\right]\sigma_{x}^{\left(1\right)}=E_{1}\left(\frac{\partial u}{\partial x}-z\frac{\partial^{2}w}{\partial x^{2}}\right)-E_{1}d_{1}H\left(t\right)$$

(7) $$\left[1-\mu \frac{\partial^{2}}{\partial x^{2}}\right]\sigma_{x}^{\left(2\right)}=E_{2}\left(\frac{\partial u}{\partial x}-z\frac{\partial^{2}w}{\partial x^{2}}\right)$$

Integrate both sides of Equations (6) and (7) with respect to *z* in the interval $\left[0,h_{1}\right]$ and $\left[-h_{2},0\right]$, using the axial force definition $F_{N}^{\left(1\right)}=b\int_{0}^{h_{1}}\sigma_{x}^{\left(1\right)}dz$ and $F_{N}^{\left(2\right)}=b\int_{-h_{2}}^{0}\sigma_{x}^{\left(2\right)}dz$, we obtain

(8) $$\;\left[1-\mu \frac{\partial^{2}}{\partial x^{2}}\right]F_{N}^{\left(1\right)}=E_{1}A_{1}\left[\frac{\partial u}{\partial x}-\frac{h_{1}}{2}\frac{\partial^{2}w}{\partial x^{2}}-d_{1}H(t)\right]$$

(9) $$\;\left[1-\mu \frac{\partial^{2}}{\partial x^{2}}\right]F_{N}^{\left(2\right)}\;=E_{2}A_{2}\left(\frac{\partial u}{\partial x}+\frac{h_{2}}{2}\frac{\partial^{2}w}{\partial x^{2}}\right)$$

Similarly, multiply both sides of Equations (6) and (7) by *z*, and integrate with respect to *z* in the interval $\left[0,h_{1}\right]$ and $\left[-h_{2},0\right]$. Using the bending moment definition $M^{\left(1\right)}=b\int_{0}^{h_{1}}z\sigma_{x}^{\left(1\right)}dz$, $M^{\left(2\right)}=b\int_{-h_{2}}^{0}z\sigma_{x}^{\left(2\right)}dz$ and the equilibrium Equations (4) and (5), the total bending moment and total shear force can be obtained:

(10) $$M=\mu (\rho_{1}A_{1}+\rho_{2}A_{2})\frac{\partial^{2}w}{\partial t^{2}}+\left(\frac{h_{1}}{2}E_{1}A_{1}-\frac{h_{2}}{2}E_{2}A_{2}\right)\frac{\partial u}{\partial x}-4\left(E_{1}I_{1}+E_{2}I_{2}\right)\frac{\partial^{2}w}{\partial x^{2}}-\frac{h_{1}}{2}E_{1}A_{1}d_{1}H(t)$$

(11) $$F_{s}=\mu (\rho_{1}A_{1}+\rho_{2}A_{2})\frac{\partial^{3}w}{\partial x\partial t^{2}}+\left(\frac{h_{1}}{2}E_{1}A_{1}-\frac{h_{2}}{2}E_{2}A_{2}\right)\frac{\partial^{2}u}{\partial x^{2}}-4\left(E_{1}I_{1}+E_{2}I_{2}\right)\frac{\partial^{3}w}{\partial x^{3}}$$

The axial force expression (8) and (9) are derived with respect to *x* and then added together; using the equilibrium Equation (3), we obtain:

(12) $$\left(E_{1}A_{1}+E_{2}A_{2}\right)\frac{\partial^{2}u}{\partial x^{2}}-\left(\frac{h_{1}}{2}E_{1}A_{1}-\frac{h_{2}}{2}E_{2}A_{2}\right)\frac{\partial^{3}w}{\partial x^{3}}=0$$

Substituting Equation (11) into equilibrium Equation (4) yields:

(13) $$\left(\frac{h_{1}}{2}E_{1}A_{1}-\frac{h_{2}}{2}E_{2}A_{2}\right)\frac{\partial^{3}u}{\partial x^{3}}-4\left(E_{1}I_{1}+E_{2}I_{2}\right)\frac{\partial^{4}w}{\partial x^{4}}=\left(\rho_{1}A_{1}+\rho_{2}A_{2}\right)\left(1-\mu \frac{\partial^{2}}{\partial x^{2}}\right)\frac{\partial^{2}w}{\partial t^{2}}$$

Taking the derivative of both sides of Equation (12) with respect to *x* and substituting it into Equation (13), we obtain the vibration control equation of the magnetostrictive composite cantilever beam considering nonlocal effects:

(14) $$\bar{EI}\frac{\partial^{4}w}{\partial x^{4}}+\bar{\rho A}\left(1-\mu \frac{\partial^{2}}{\partial x^{2}}\right)\frac{\partial^{2}w}{\partial t^{2}}=0$$

where $\bar{EI}$ is the equivalent flexural stiffness, $\bar{\rho A}$ is the equivalent inertial mass, and $\bar{EA}$ is equivalent tensile stiffness of the composite beam; their expressions are as follows:

$$\bar{EI}=E_{1}I_{1}+E_{2}I_{2}+{\left(\frac{h}{2}\right)}^{2}\bar{EA},\;\;\bar{\rho A}=\rho_{1}A_{1}+\rho_{2}A_{2},\;\;\bar{EA}=\frac{1}{1/E_{1}A_{1}+1/E_{2}A_{2}}$$

It should be noted that in the expression of the equivalent flexural stiffness $\bar{EI}$, in addition to the direct superposition of the flexural stiffness of the two layers of the beam, there is also an additional contribution from the equivalent tensile stiffness $\bar{EA}$. This is because the total deformation of the double-layer composite beam under the action of the magnetic field is the coupling deformation of axial stretching and transverse bending, which accords with the deformation mechanism of the magnetostrictive cantilever beam resonator. In addition, the nonlocal parameter *μ* does not affect the stiffness and inertial mass of the beam [27].

At the fixed end of the cantilever beam (*x* = 0, $-h_{2}\leq z\leq h_{1}$), the following boundary condition of zero displacement should be satisfied:

(15) $$\left\{\begin{array}{l} u_{x}^{\left(i\right)}\left(0,z,t\right)=u(0,t)-z\frac{\partial w\left(0,t\right)}{\partial x}=0\\ u_{z}^{\left(i\right)}\left(0,z,t\right)=0 \end{array}\right.$$

At the free-end (*x* = *L*), the boundary condition for free stress is usually not exactly satisfied; since we propose the Saint-Venant boundary condition, i.e., the internal force is zero, we have:

(16) $$\left\{\begin{array}{l} {\left.\left(F_{N}^{\left(1\right)}+F_{N}^{\left(2\right)}\right)\right|}_{x=L}=0\\ {\left.F_{S}\right|}_{x=L}=0\\ {\left.M\right|}_{x=L}=0 \end{array}\right.$$

By integrating both sides of Equation (12) with respect to *x*, and using the fixed end boundary conditions (15) and the free end boundary conditions (16), the axial displacement of the interface can be written as follows:

(17) $$u\left(x,t\right)=\frac{E_{1}A_{1}h_{1}-E_{2}A_{2}h_{2}}{2\left(E_{1}A_{1}+E_{2}A_{2}\right)}\frac{\partial w\left(x,t\right)}{\partial x}+\frac{E_{1}A_{1}}{E_{1}A_{1}+E_{2}A_{2}}d_{1}H\left(t\right)x$$

Substituting Equation (17) into Equations (10) and (11), the bending moment and shear force of the composite beam section can be expressed as follows:

(18) $$M=-\bar{EI}\frac{\partial^{2}w}{\partial x^{2}}+\mu \;\bar{\rho A}\frac{\partial^{2}w}{\partial t^{2}}-\frac{h}{2}\bar{EA}d_{1}H\left(t\right)$$

(19) $$F_{S}=-\bar{EI}\frac{\partial^{3}w}{\partial x^{3}}+\mu \bar{\rho A}\frac{\partial^{3}w}{\partial x\partial t^{2}}$$

From Equations (17)–(19), we obtain the boundary conditions of the magnetostrictive composite cantilever beam as follows:

(20) $$\begin{array}{l} w\left(0,t\right)=0,\;\frac{\partial w\left(0,t\right)}{\partial x}=0\\ {\left[-\bar{EI}\frac{\partial^{2}w}{\partial x^{2}}+\mu \bar{\rho A}\frac{\partial^{2}w}{\partial t^{2}}\right]}_{x=L}=\frac{h}{2}\bar{EA}d_{1}H\left(t\right)\\ {\left[-\bar{EI}\frac{\partial^{3}w}{\partial x^{3}}+\mu \bar{\rho A}\frac{\partial^{3}w}{\partial x\partial t^{2}}\right]}_{x=L}=0 \end{array}$$

For the practical application of the resonators, it is usually necessary to consider the steady-state periodic vibration of cantilever beam elements caused by periodic magnetic field excitation, so that their displacement response satisfies the following periodic conditions:

(21) $$w\left(x,0\right)=w\left(x,T\right),\;\frac{\partial w\left(x,0\right)}{\partial t}=\frac{\partial w\left(x,T\right)}{\partial t}$$

where 0≤x≤L, *T* = 2π/*ω* and *ω* are the vibration period and relative circular frequency respectively.

Up to now, the vibration problem of the magnetostrictive composite cantilever resonator considering the nonlocal effect is mathematically reduced to solving the governing Equation (14) associated with the boundary conditions (20) and periodic conditions (21). Different from Euler’s classical beam theory, the vibration equation and boundary conditions contain the nonlocal parameter *μ* that describes nonlocal effects, and when μ=0, the vibration governing equation degenerates to the classical Euler beam vibration governing equation without considering nonlocal effects. It is worth noting that the vibrational excitation term does not appear in the vibration equation, but in the boundary conditions.

## 3. Solution to the Vibration Problem

The magnetic field *H* is assumed to be a harmonic function of time *t*; that is, $H\left(t\right)=H_{0}e^{i\omega t}$($i=\sqrt{-1}$). Denote the dimensionless variables and parameters as follows:

(22) $$\xi =\frac{x}{L},\;\tau =\frac{t}{T}=\frac{\omega t}{2\pi },\;l=\frac{\sqrt{\mu }}{L},\;\beta =\frac{\bar{\rho A}L^{4}}{\bar{EI}},\;\gamma =\frac{h\bar{EA}L}{2\bar{EI}},\;v\left(\xi ,\tau \right)=\frac{w\left(x,t\right)}{L}$$

We can rewrite the governing Equation (14) as follows:

(23) $$\frac{\partial^{4}v}{\partial \xi^{4}}+\frac{\beta \omega^{2}}{4\pi^{2}}\left(1-l^{2}\frac{\partial^{2}}{\partial \xi^{2}}\right)\frac{\partial^{2}v}{\partial \tau^{2}}=0$$

Accordingly, the dimensionless form of the definite solution condition (20) can be rewritten as follows:

(24a) $$v\left(0,\tau \right)=0,\;{\left.\frac{\partial v}{\partial \xi }\right|}_{\xi =0}=0$$

(24b) $${\left[-\frac{\partial^{2}v}{\partial \xi^{2}}+l^{2}\frac{\beta \omega^{2}}{4\pi^{2}}\frac{\partial^{2}v}{\partial \tau^{2}}\right]}_{\xi =1}=\gamma d_{1}\bar{H}\left(\tau \right)$$

(24c) $${\left[-\frac{\partial^{3}v}{\partial \xi^{3}}+l^{2}\frac{\beta \omega^{2}}{4\pi^{2}}\frac{\partial^{3}v}{\partial \xi \partial \tau^{2}}\right]}_{\xi =1}=0$$

where $\bar{H}\left(\tau \right)=H_{0}e^{i2\pi \tau }$. The dimensionless form of the periodic condition (21) is as follows:

(24) $$v\left(\xi ,0\right)=v\left(\xi ,1\right),\frac{\partial v\left(\xi ,0\right)}{\partial \tau }=\frac{\partial v\left(\xi ,1\right)}{\partial \tau }$$

Based on the periodic condition (25), the magnetoelastic vibration response of the composite beam can be expressed as the following separate variable form:

(25) $$v\left(\xi ,\tau \right)=W\left(\xi \right)e^{i2\pi \tau }$$

where $W\left(\xi \right)$ is the displacement amplitude. Substituting Equation (26) into Equation (23) and eliminating the time variable *τ*, the following ordinary differential equation with constant coefficients is obtained:

(26) $$\frac{d^{4}W\left(\xi \right)}{d\xi^{4}}+l^{2}\beta \omega^{2}\frac{d^{2}W\left(\xi \right)}{d\xi^{2}}-\beta \omega^{2}W\left(\xi \right)=0$$

The general solution of Equation (27) is given in the form:

(27) $$W\left(\xi \right)=B_{1}\cosh\left[\lambda_{1}(\omega )\xi \right]+B_{2}\sinh\left[\lambda_{1}(\omega )\xi \right]+B_{3}\cos\left[\lambda_{2}(\omega )\xi \right]+B_{4}\sin\left[\lambda_{2}(\omega )\xi \right]$$

where

$$\lambda_{1}\left(\omega \right)=\frac{1}{\sqrt{2}}{\left\{\sqrt{{\left(l^{2}\beta \omega^{2}\right)}^{2}+4\beta \omega^{2}}-l^{2}\beta \omega^{2}\right\}}^{\frac{1}{2}},\;\lambda_{2}\left(\omega \right)=\frac{1}{\sqrt{2}}{\left\{\sqrt{{\left(l^{2}\beta \omega^{2}\right)}^{2}+4\beta \omega^{2}}+l^{2}\beta \omega^{2}\right\}}^{\frac{1}{2}}$$

Substituting the solution expression Equation (28) into the boundary Equations (24a)–(24c), we obtain the system of linear algebraic equations for determining the coefficients *B_j_* (*j* = 1, 2, 3, 4) as follows:

(28) $$\left[\begin{array}{cccc} 1 & 0 & 1 & 0\\ 0 & \lambda_{1}\left(\omega \right) & 0 & \lambda_{2}\left(\omega \right)\\ \lambda_{2}^{2}\left(\omega \right)\mathrm{ch}\lambda_{1}\left(\omega \right) & \lambda_{2}^{2}\left(\omega \right)\mathrm{sh}\lambda_{1}\left(\omega \right) & -\lambda_{1}^{2}\left(\omega \right)\cos\lambda_{2}\left(\omega \right) & -\lambda_{1}^{2}\left(\omega \right)\sin\lambda_{2}\left(\omega \right)\\ \lambda_{2}\left(\omega \right)\mathrm{sh}\lambda_{1}\left(\omega \right) & \lambda_{2}\left(\omega \right)\mathrm{ch}\lambda_{1}\left(\omega \right) & \lambda_{1}\left(\omega \right)\sin\lambda_{2}\left(\omega \right) & -\lambda_{1}\left(\omega \right)\cos\lambda_{2}\left(\omega \right) \end{array}\right]\left[\begin{array}{c} B_{1}\\ B_{2}\\ B_{3}\\ B_{4} \end{array}\right]=\left[\begin{array}{c} 0\\ 0\\ \gamma d_{1}H_{0}\\ 0 \end{array}\right]$$

Denote the coefficient matrix of (29) as **A**(*ω*).

For the case of excited vibration $H\left(\tau \right)\neq 0$, that is, the closed circuit boundary conditions, we consider the case of periodic vibration: the magnetostrictive layer is subjected to a uniform magnetic field of sinusoidal variation with time, that is $H\left(\tau \right)=H_{0}\sin2\pi \tau$. By substituting the known excitation frequency *ω* into Equation (29), the coefficient *B_j_* can be determined, and then using Equation (26), we finally obtain the vibration response of the composite beam.

For the case of free vibration, the external excited magnetic field $H\left(\tau \right)\equiv 0$, that is the open-circuit boundary conditions, the magnetic induction on the surfaces of the beam is zero; hence the boundary condition (29) becomes homogeneous. The condition $B_{j}\neq 0$ from Equation (29) gives det(A(ω))=0. Thus, the natural frequency equation of the composite cantilever beam can be derived as follows:

(29) $$\lambda_{1}^{4}+\lambda_{2}^{4}+2\lambda_{1}^{2}\lambda_{2}^{2}\mathrm{ch}\lambda_{1}\cos\lambda_{2}+\lambda_{1}\lambda_{2}\left(\lambda_{2}^{2}-\lambda_{1}^{2}\right)\mathrm{sh}\lambda_{1}\sin\lambda_{2}=0$$

Substituting the expression of $\lambda_{i}\left(\omega \right)$ (*i* = 1, 2) into Equation (30), yields

(30) $$l^{2}\left(l^{2}\beta \omega^{2}+\sqrt{\beta \omega^{2}}\mathrm{sh}\lambda_{1}\sin\lambda_{2}\right)+2\mathrm{ch}\lambda_{1}\cos\lambda_{2}=-2$$

The natural frequencies of each order $f_{n}=\omega_{n}/2\pi \left(n=1,2\cdots \right)$ are obtained from Equation (31). Moreover, by means of the normalization condition $\sum_{j=1}^{4}B_{j}^{2}=1$, the coefficients *B_j_* (*j* = 1, 2, 3, 4) corresponding to $\lambda_{i}\left(\omega \right)$ can be obtained from Equation (29). Consequently, the vibration mode function $W_{n}\left(\xi \right)$ is given by Equation (28).

## 4. Numerical Examples and Discussion

We now apply our solution to a double-layer beam composed of the magnetostrictive (Tb_0.3_Dy_0.7_)_0.42_ Fe_0.58_ and silicon to investigate the influence of the nonlocal parameter on natural frequency and mode shapes. Take the magnetostrictive composite cantilever beam device mentioned in reference [17] as an example, which has a length *L* = 20 mm, width *b* = 5 mm, and a total thickness *h* = 60 μm. For the magnetostriction layer, the thickness *h*_1_ = 10 μm, the elastic modulus *E*_1_ = 50 Gpa, the density *ρ*_1_ = 9.25 × 10^3^ kg/m^3^, the Poisson’s ratio *ν*_1_ = 0.3, and the magnetostriction coefficient *d*_1_ = 5.027 × 10^−8^ m/A. For the substrate layer, the thickness *h*_2_ = 10 μm, the elastic modulus *E*_2_ = 130 Gpa, the density *ρ*_2_ = 2.33 × 10^3^ kg/m^3^ and Poisson’s ratio *ν*_2_ = 0.28.

By referring to the value of the dimensionless nonlocal parameter *l* in reference [27], we consider the parameter *l* to be 0.05, 0.1, 0.15, 0.2, 0.25, 0.3, 0.35 and 0.4 in this paper. The first three natural frequencies for the composite beam with different parameters *l* calculated by Equation (30) are shown in Table 1 and compared with the results without nonlocal effects (when *l* = 0, the nonlocal composite beam is reduced to the classical one). It is found that, with increasing nonlocal parameter *l* and the order of modes, the natural frequency differences between, with, and without nonlocal effects increase monotonically. For example, when the parameter *l* = 0 and *l* = 0.2, the relative deviations of natural frequencies are 1.86% for *f*_1_, 6.14% for *f*_2_ and 40.3% for *f*_3_. Obviously, the nonlocal effect has a greater impact on the higher order frequencies than the lower-order ones. In the practical application of micro/nano electromechanical systems, magnetostrictive resonators are more likely to exhibit high-frequency vibration characteristics, so the influence of nonlocal effects on their frequency cannot not be ignored.

**Figure 2:**
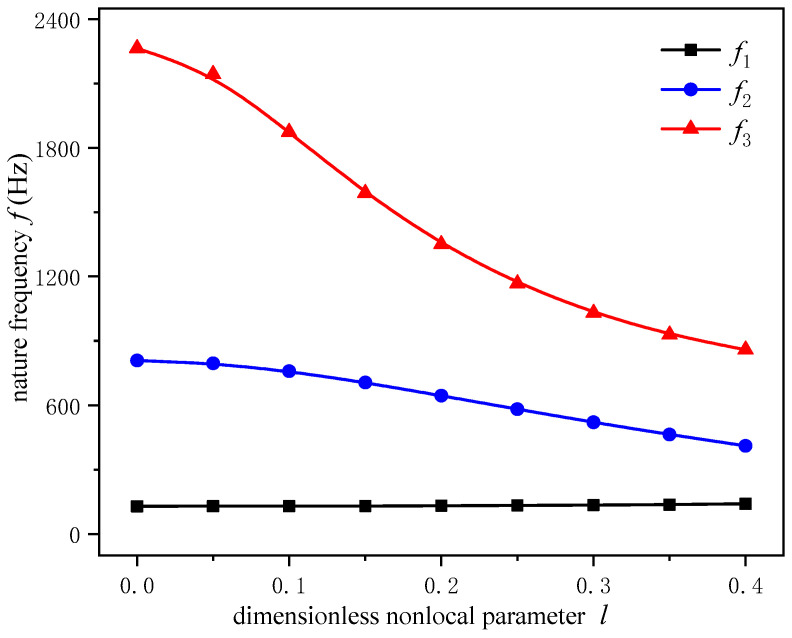
The first three orders of nature frequencies of the composite cantilever beam vary with the dimensionless nonlocal parameters.

Figure 2 shows the variation curves of the first three orders of natural frequencies for the composite beam with the dimensionless nonlocal parameter *l*. The fundamental frequency is the first natural frequency of the structure. For sensors and actuators, the fundamental frequency is the most easily excited vibration frequency of the structure, and so it is one of the important vibration indicators. It is interesting that with the increasing parameter *l*, the fundamental frequency of the composite beam increases, while the second and third natural frequencies decrease. That is, the frequency bandwidths of the composite beam become narrower, and the narrowing speed accelerates with the increase in the parameter *l*. This demonstrates that the nonlocal effect weakens the effective structural stiffness of the composite beam and thus causes the frequency shift effect, which affects the vibration characteristics of the resonator.

**Figure 3:**
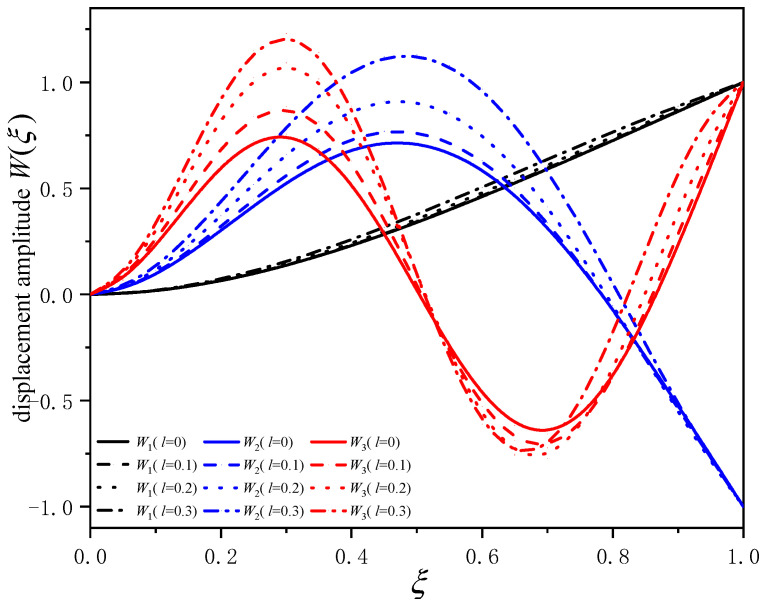
Curves of first three orders of vibration modes of composite beam with different nonlocal parameters (*h*_1_/*h* = 0.167).

Figure 3 gives the first three orders of mode shape curves of the composite beam with different dimensionless nonlocal parameters *l*. It is observed that the mode shapes roughly follow three groups. The solid line of each group curve represents the mode shape when the parameter *l* = 0, and the dashed line represents the corresponding mode curve when the parameter *l* is 0.1, 0.2 and 0.3. It can be seen that for the same-order mode, the larger the nonlocal parameter *l*, the greater the change in beam amplitude and the more obvious the vibration effect. It is worth noting that nonlocal effects could also cause changes in the positions where the amplitude of the beam is zero (i.e., the modal node). Taking the second-order mode (blue curves) as an example, the modal node position moved from ξ=0.75 for *l* = 0 towards the free end to about ξ=0.8 for *l* = 0.3. Therefore, in the practical application of resonators, the position errors of modal nodes and antinodes (i.e., where amplitude is at its peak) would affect the function of high-performance devices. In other words, both modal node and anti-node positions could be tuned by the nonlocal parameter, which could be very beneficial to the functional design of composite micro beam devices.

In order to further demonstrate the effect of the nonlocal parameter on the natural frequency of a composite cantilever beam with different transverse dimensions, the first three orders of natural frequencies with different layer thickness ratios c($c=h_{1}/h$) and nonlocal parameters *l* are listed in Table 2 for a fixed total thickness of the composite beam *h*. Here, not only is the natural frequency determined, but the shift in the natural frequency is also studied. We introduce the frequency difference and the relative frequency shift, noted as $\Delta f_{j}=f_{j}-f_{0j}$ and $R_{j}=\left|\Delta f_{j}/f_{0j}\right|\cdot 100\%$ (j=1,2,3), where $f_{j}$, $f_{0j}$ are frequencies with and without nonlocal effects, respectively. It is observed clearly that, as the thickness of the magnetostrictive layer increases (that is, *c* increases), the natural frequencies decrease, and the frequency difference $\Delta f_{j}$ also decreases, but the relative frequency shift $R_{j}$ almost does not change with the thickness ratio *c*.

**Figure 4:**
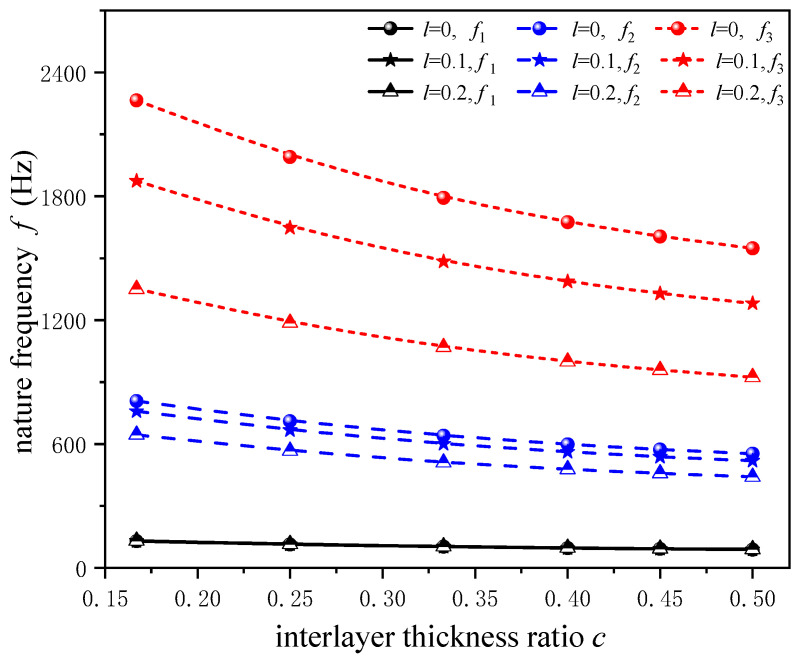
Nature frequencies *f* versus interlayer thickness ratios *c* with different microscale parameters *l.*

Figure 4 shows the variation curves of the natural frequency of the composite cantilever beam with thickness ratio *c* when the nonlocal parameter *l* = 0, 0.1, 0.2. It can be seen that, when the magnetostrictive layer is thicker (the larger *c* is), the overall stiffness of the composite beam is smaller, and the natural frequency of the structure is lower. In addition, it is clear that the difference in frequency shift caused by nonlocal effects is larger for higher vibration modes.

**Figure 5:**
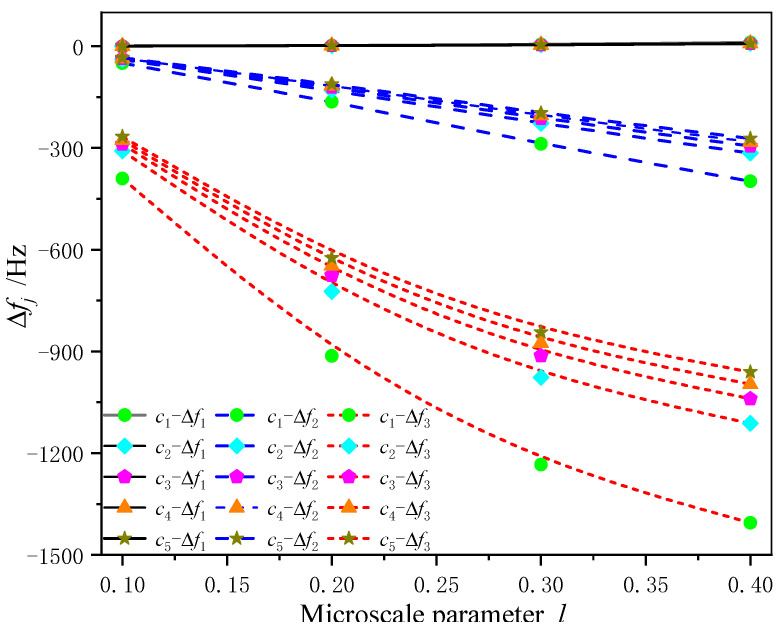
Frequency differences $\Delta f_{j}$ versus nonlocal parameters *l* with different transverse dimensions.

**Figure 6:**
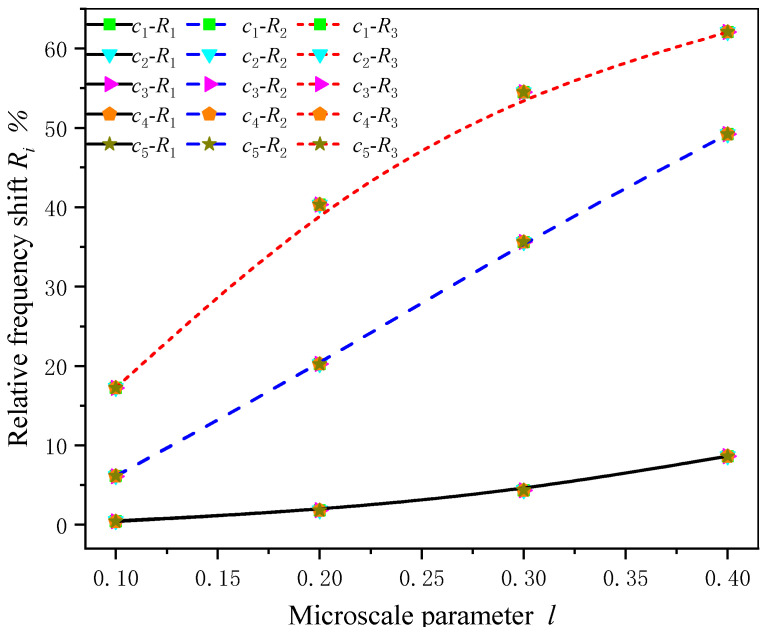
Relative frequency shifts $R_{j}$ versus nonlocal parameters *l* with different transverse dimensions.

Figure 5 and Figure 6 show the first three natural frequency difference and relative frequency shift curves of the composite beam with different transverse dimensions as a function of nonlocal parameters. With the decrease in the interlayer thickness ratio and the increase in the microscale parameters, the frequency difference $\Delta f_{j}$ with and without nonlocal effects presents a more nonlinear difference, while the thickness ratio has little influence on the relative frequency shift $R_{j}$ for the same mode and almost no change. This feature indicates that, for a given composite beam thickness, a slight adjustment to the nonlocal parameter *l* would not influence the relative frequency shift of the structure, regardless of the layer thickness of the two materials.

**Figure 7:**
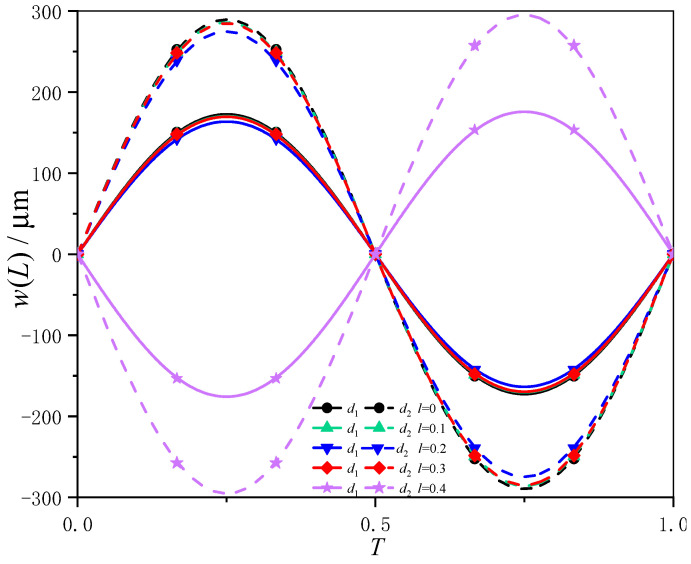
The deflection response curves on free end of composite cantilever beam with different magnetostrictive coefficient *d* varies with microscale parameter *l* (*c* = 0.167).

In order to further demonstrate the effect of the nonlocal parameter on the output of the magnetostrictive resonator, Figure 7 shows the deflection response curves (at one period *T*) on the free end of the magnetostrictive thin-film cantilever device [17] with different nonlocal parameters subject to the external excitation frequency *H*_0_ = 500 Hz. The curves of the solid line group represent the magnetostrictive coefficient $d_{1}=5.027\times 10^{-8}\;m/A$, and the curves of the dashed line group represent the magnetostrictive coefficient $d_{2}=8.437\times 10^{-8}\;m/A$ in Figure 7. It can be seen from the figure that the magnetostrictive coefficient *d* has a significant influence on the output of deflection for the free end, while the nonlocal parameter *l* has a slight effect on the deflection of the free end.

## 5. Conclusions

The vibration problem of the magnetostrictive composite cantilever beam resonator is investigated using nonlocal elastic and Euler beam theory. The frequency equation and vibration mode function of the composite magnetostrictive cantilever are obtained by means of the separation of variables method and the analytic solution of ordinary differential equations. The effects of nonlocal parameters, interlayer thickness, and magnetostriction coefficient on the vibration characteristics of the magnetostrictive resonator are investigated numerically.

1. Different from the classical Euler beam theory, the vibration equation and boundary conditions of the nonlocal theory contain the parameter μ to describe the nonlocal effects, and when μ = 0, the vibration equation and boundary conditions degenerate to the classical theoretical form (without considering nonlocal effects).
2. The coupling deformation of axial stretching and transverse bending occurs in a double-layer composite beam subjected to the magnetic field, and the vibration governing equation is a fourth-order ordinary differential equation. In particular, the equivalent flexural stiffness of the beam includes not only the flexural stiffness but also the tensile stiffness, while the nonlocal parameter does not affect the stiffness and inertial mass of the beam.
3. The natural frequencies and vibration modal shapes of magnetostrictive composite beams depend strongly on the nonlocal parameters. Consequently, the nonlocal effects reinforce the vibration characteristics of the micro/nano resonator, especially on the high-order vibration modes.
4. Compared to the vibration modes, the nonlocal parameter l has only a slight effect on the deflections (i.e., elastic displacements) of the free end. The significant influence of l on natural frequency and vibration modes in different thickness laminated beams could be utilized in designing better laminated micro/nano magnetostrictive resonators with nonlocal effects.

## Figures and Tables

**Table 1:** The first three order nature frequencies of the composite cantilever beam with different dimensionless nonlocal parameters *l* (Hz).

	*l =* 0	*l =* 0.05	*l =* 0.1	*l =* 0.15	*l =* 0.2	0.25	*l =* 0.3	0.35	*l =* 0.4
*f*_1_	129.0 *	129.17	129.6	130.3	131.4	132.8	134.6	137.0	140.2
*f*_2_	808.7 *	795.5	758.9	706.13	645.0	581.87	520.6	463.4	410.8
*f*_3_	2264.2 *	2144.4	1874.0	1589.0	1351.0	1167.7	1030.4	929.1	858.6

Note: Data marked with asterisk ‘*’ are the results of *l* = 0, which are consistent with the natural frequency results of classic Euler beam theory in reference [16].

**Table 2:** First three orders of nature frequencies and relative frequency shift rates of composite beam with different interlayer thickness ratios *c.*

*c*	*l*	f_i_/Hz			$\Delta f_{i}$/Hz			R_i_/%		
		*f*_1_	*f*_2_	*f*_3_	$\Delta f_{1}$	$\Delta f_{2}$	$\Delta f_{3}$	*R*_1_	*R*_2_	*R*_3_
0.167	0	129.03	808.63	2264.21	/	/	/	/	/	/
	0.1	129.59	758.92	1873.98	0.56	−49.71	−390.23	0.43	6.15	17.23
	0.2	131.36	645.01	1350.99	2.33	−163.62	−913.22	1.81	20.23	40.33
	0.3	134.63	520.64	1030.38	5.6	−287.99	−1233.83	4.34	35.61	54.49
	0.4	140.16	410.76	858.64	11.13	−397.87	−1405.57	8.63	49.20	62.08
0.333	0	102.14	640.11	1792.33	/	/	/	/	/	/
	0.1	102.63	600.75	1483.42	0.49	−39.36	−308.91	0.48	6.15	17.24
	0.2	103.98	510.57	1069.44	1.84	−129.54	−722.89	1.80	20.24	40.33
	0.3	106.58	412.13	815.65	4.44	−227.98	−976.68	4.35	35.62	54.49
	0.4	110.96	325.14	679.7	8.82	−314.97	−1112.63	8.64	49.21	62.08
0.4	0	95.46	598.2	1675	/	/	/	/	/	/
	0.1	95.87	561.74	1386.3	0.41	−36.46	−288.7	0.43	6.09	17.24
	0.2	97.2	477.15	999.43	1.74	−121.05	−675.57	1.82	20.24	40.33
	0.3	99.6	385.16	762.25	4.14	−213.04	−912.75	4.34	35.61	54.49
	0.4	103.69	303.87	635.22	8.23	−294.33	−1039.78	8.62	49.20	62.08
0.45	0	91.49	573.33	1605.37	/	/	/	/	/	/
	0.1	91.87	538.09	1328.7	0.38	−35.24	−276.67	0.42	6.15	17.23
	0.2	93.14	457.32	957.88	1.65	−116.01	−647.49	1.80	20.23	40.33
	0.3	95.46	369.14	730.57	3.97	−204.19	−874.8	4.34	35.61	54.49
	0.4	99.38	291.23	608.8	7.89	−282.1	−996.57	8.62	49.20	62.08
0.5	0	88.22	552.85	1548	/	/	/	/	/	/
	0.1	88.6	518.85	1281.2	0.38	−34	−266.8	0.43	6.15	17.24
	0.2	89.81	440.99	923.65	1.59	−111.86	−624.35	1.80	20.23	40.33
	0.3	92.05	355.94	704.46	3.83	−196.91	−843.54	4.34	35.62	54.49
	0.4	95.82	280.81	587.04	7.6	−272.04	−960.96	8.61	49.21	62.08

## Data Availability

Data are contained within the article.
